# A case of hypertrophic cardiomyopathy combined with muscular ventricular septal defect and abnormal origin of right coronary artery

**DOI:** 10.1186/s12872-018-0997-8

**Published:** 2019-01-14

**Authors:** Guang-mei Zheng, Jiao Bai, Jun-ming Tang, Fang-cheng Zhu, Hong-xia Jing

**Affiliations:** 1 Department of ultrasound, Renmin Hospital, Hubei University of Medicine, Shiyan, 442000 Hubei China; 2 Institute of clinical medicine, Renmin Hospital, Hubei University of Medicine, Shiyan, 442000 Hubei China; 3 Department of Forensic Medicine, Hubei University of Medicine, Shiyan, 442000 Hubei China

**Keywords:** Hypertrophic cardiomyopathy, Ventricular septal defect, Right coronary artery, Transthoracic echocardiography, Computed tomography angiography

## Abstract

**Background:**

Hypertrophic cardiomyopathy (HCM) is a myocardial disease. However, the coexistence of HCM with muscular ventricular septal defect (VSD), especially those with both incomplete spontaneous closure and coronary abnormal origin, is relatively rare.

**Case presentation:**

We report herein a unique case of HCM accompanied with incomplete spontaneous closure of muscular VSD and abnormal origin of right coronary artery (RCA) in a 26-year-old man, which was diagnosed by combination of transthoracic 2-dimensional (2D), color Doppler, Contrast-enhanced echocardiography and computed tomography angiography (CTA).

**Conclusions:**

To our knowledge, this is the first report that HCM along with the incomplete spontaneous closure of muscular VSD and anomalous RCA arising from left coronary sinus was revealed through combination of transthoracic 2D, color Doppler, Contrast-enhanced echocardiography and CTA. These observations indicated that other associated anomalies in patients with HCM could be easily missed if examined by the single echocardiography. Therefore, HCM-associated congenital abnormalities should be screened by combination of transthoracic 2D, color Doppler, contrast-enhanced echocardiography, and CTA.

**Electronic supplementary material:**

The online version of this article (10.1186/s12872-018-0997-8) contains supplementary material, which is available to authorized users.

## Background

Individual hypertrophic cardiomyopathy (HCM), muscular ventricular septal defect (VSD), and abnormal origin of coronary artery are common congenital heart diseases. Indeed, HCM and coronary artery anomalies have been recognized as the leading causes of exercise-related sudden cardiac death, especially among the young people. Recently, a case of HCM associated with VSD has been reported [1]. And several cases of coexistences of HCM with anomalous origin of coronary artery have also been reported [2–4]. However, concurrency of three different types of abnormalities has not been reported.

## Case presentation

**Fig. 1:**
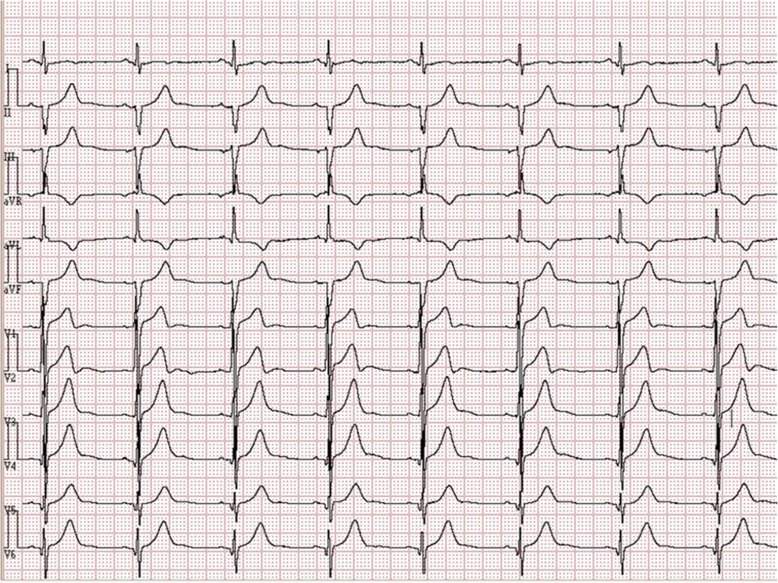
12-lead ECG revealed sinus bradycardia, left anterior fascicular block, T-wave anomaly and abnormal Q wave on the leads of left ventricle anterolateral wall

A 26-year-old man was admitted to our hospital due to atypical chest pain persisted for many years. He was diagnosed as HCM in another hospital two years ago, and had received medical therapy (angiotensin-converting enzyme inhibitors and Beta blockers) for 18 months. Physical examinations did not show abnormality. A 12-lead electrocardiogram (ECG) showed sinus bradycardia, left anterior fascicular block, T-wave anomaly, and abnormal Q wave on the leads of left ventricular anterolateral wall (Fig. 1).

**Fig. 2:**
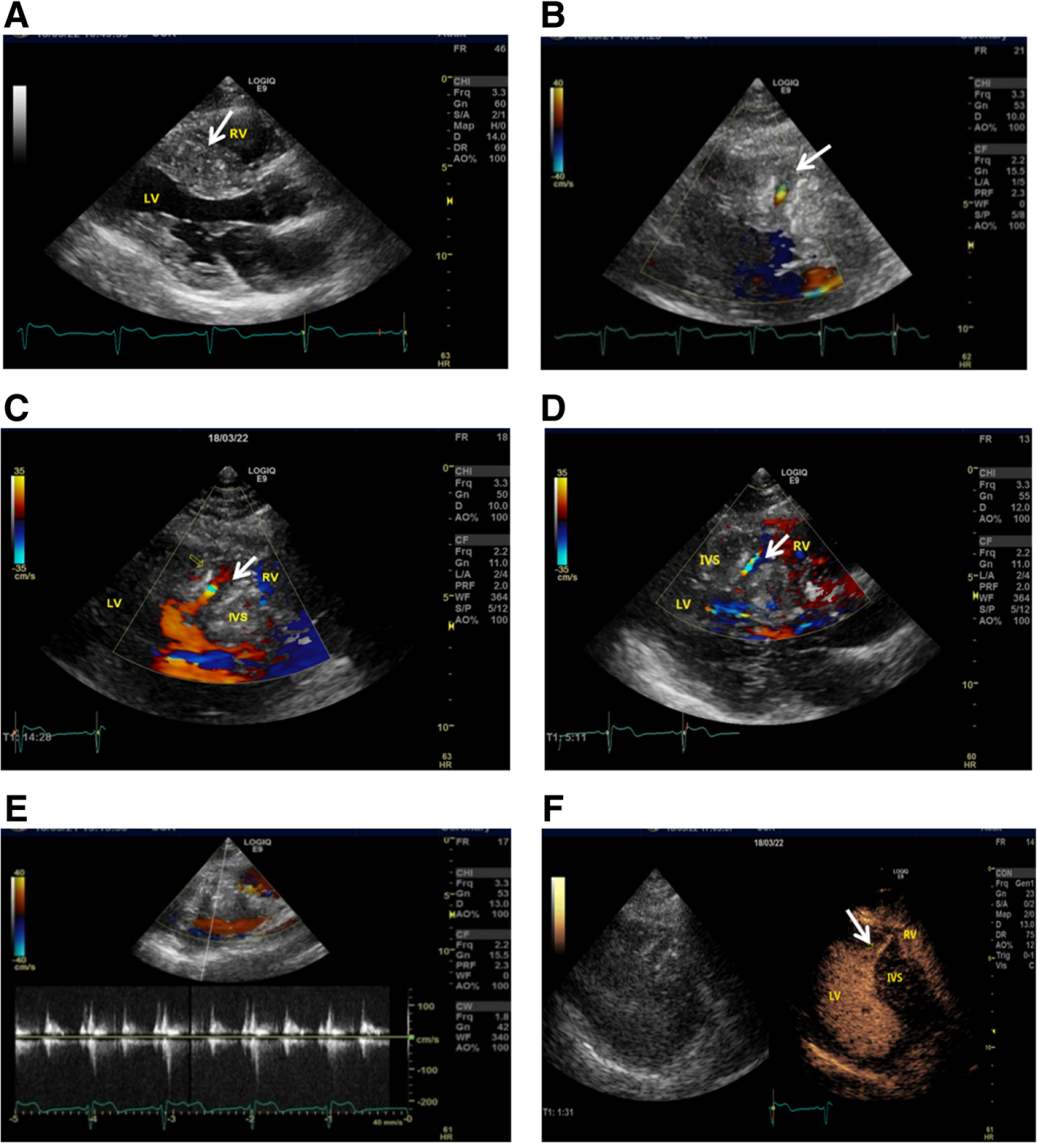
Representative images of hypertrophic interventricular septum, muscular VSD and anomalous blood flow signals by TTE. **a** Hypertrophic interventricular septum (white arrow). **b** Non-standard parasternal view showing small muscular defect with the echo enhancement of the broken end and blood flow in systolic phase (white arrow). **c** Systolic blood flow (white arrow) from left ventricle into the interventricular myocardium. **d** Systolic blood flow (white arrow) from interventricular myocardium into the left ventricle. **e** Image of a peak flow speed of 1.1 to 1.3 m/s during the systolic phase. **f** Contrast-enhanced echocardiography showed small defects (white arrow) being interlinked with the left ventricular cavity, but not with the right ventricular cavity. LV: left ventricle; RV: right ventricle; IVS: interventricular septum

2-dimensional(2D) transthoracic echocardiography(TTE)indicated hypertrophy (21 mm in diastolic phase) in the interventricular septum (Fig. 2a; Additional file 1: Movie 1). There were no detectable gradients with Doppler echocardiography in the left ventricular outflow tract at rest. However, a small defect with echo enhancement of the broken end was observed within the hypertrophic interventricular septum (Fig. 2b). Doppler echocardiography showed systolic blood flow in a specific direction from left ventricle into the interventricular myocardium (Fig. 2c, Additional file 2: Movie 2) with a peak flow speed of 1.1 to 1.3 m/s during the systolic phase (Fig. 2e). In turn, the blood flow in the opposite direction had a similar flow speed (Fig. 2d, Additional file 2: Movie 2). Contrast-enhanced echocardiography further indicated that the small defect was interlinked with the left ventricular cavity, but not with the right ventricular cavity (Fig. 2f; Additional file 3: Movie 3).

**Fig. 3:**
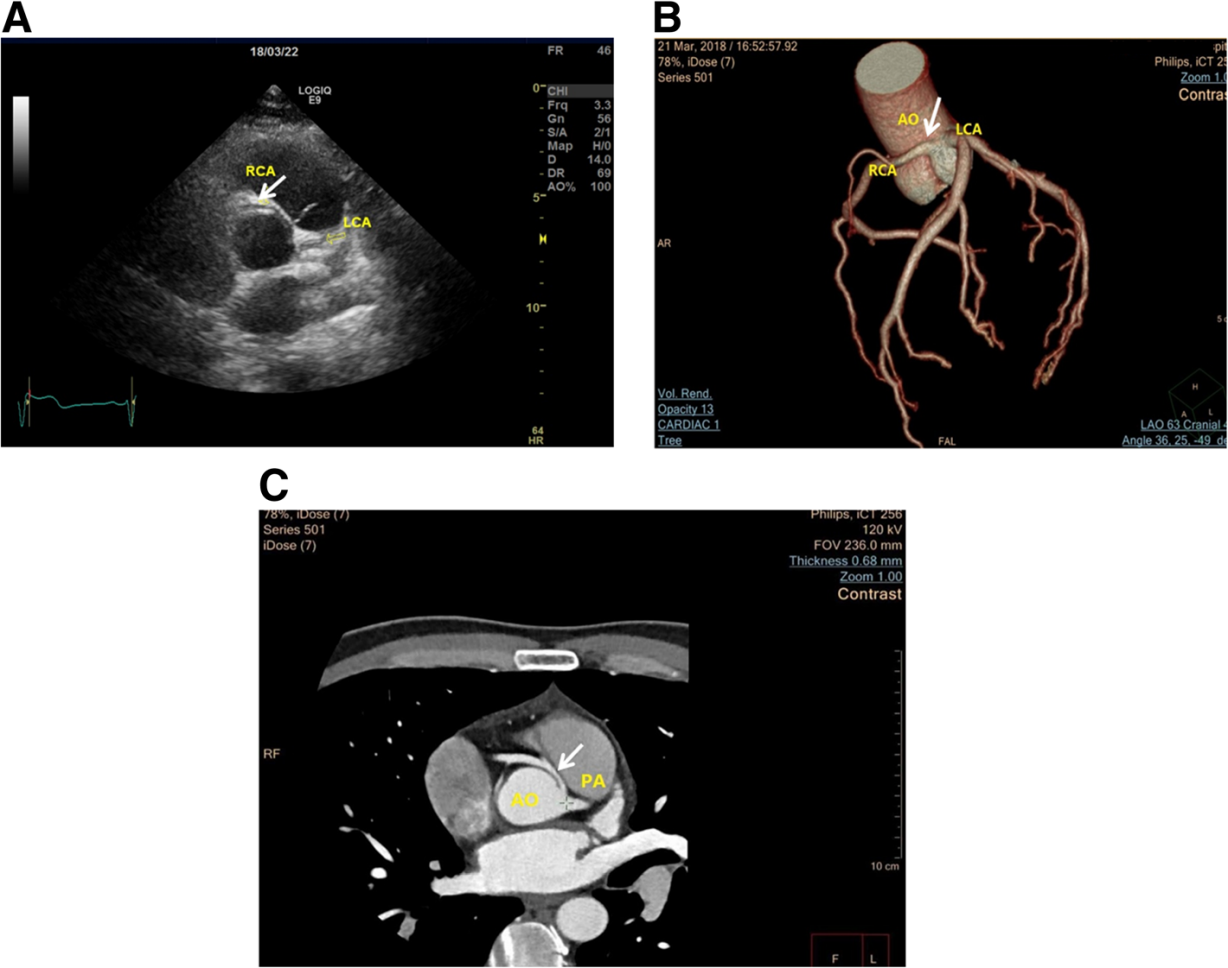
The RCA arising from left sinus of Valsalva in the patient. **a** Parasternal short-axis view of TTE, the anomalous RCA (white arrow) origin from the left sinus of Valsalva and intramural course of anomalous RCA between the aorta and pulmonary artery. **b-c** Representative CTA images of the patient. CTA showed RCA from left sinus of Valsalva (B, white arrow), and it had an interarterial course between the pulmonary trunk and aorta (C, white arrow). AO: aorta; PA: pulmonary artery; LCA: left coronary artery


In addition, the anomalous RCA originating from left sinus of Valsalva was observed using 2D TTE (Fig. 3a, Additional file 4: Movie 4). CTA further confirmed that RCA arised from left sinus of Valsalva with an interarterial course between the aorta and pulmonary artery (Fig. 3b and c).

Additional file 1: Movie 1. TTE revealed the hypertrophic interventricular septum in the parasternal left ventricle long-axis view. (WMV 905 kb)



Additional file 2: Movie 2. TTE revealed the systolic blood flow signals representing a muscular VSD within the hypertrophic ventricular septum. (WMV 469 kb)



Additional file 3: Movie 3. Contrast-enhanced echocardiography showed the small defect only being interlinked with the left ventricular cavity, but not with the right ventricular cavity. (WMV 894 kb)



Additional file 4: Movie 4. TTE revealed the RCA arising from left sinus of Valsalva. (WMV 740 kb)


Syncope or family history of sudden cardiac death was not identified. But the patient’s mother, a 53-year-old woman, had been diagnosed with ventricular septal hypertrophy (17 mm in diastolic phase) in our department. Considering the expensive cost, the patient and his mother declined the genetic testing. The young patient was advised to have regular ultrasound examination and avoid intensive physical exercise.

## Discussion and conclusions

HCM, VSD, and abnormal origin of coronary artery are common congenital heart diseases. Although HCM with VSD and HCM with anomalous origin of coronary artery have been reported [1–4], concurrencies of three types of abnormalities are rare. Our present case report provides the evidence for the coexistences of HCM, VSD and coronary artery anomalies.

Published case reports show higher prevalence of VSD with HCM in children than adult [5]. Only one case of VSD with HCM is reported in a 24-year-old female [1]. Actually, the systolic blood flow signals representing a muscular VSD have been identified within the hypertrophic ventricular septum, which was different from a coronary artery-left ventricular fistulae characterized by diastolic blood flow signals [6]. However, in our patient, the systolic blood flow speed was not as fast as the typical VSD. The unique dual direction blood flow between left ventricle and the interventricular myocardium were found using Doppler echocardiography, promoting us to further observe the difference of the patient’s VSD from common VSD. Following contrast-enhanced echocardiography, the unique small defect, i.e., the interlink with the left ventricular cavity, but not the right ventricular cavity, is very different from most of the small muscular VSDs that can close spontaneously within the first two years of life [7], suggesting that some unknown factors may be involved in the process of incomplete spontaneous closure of muscular VSD. Unfortunately, we are unable to establish any genetic association because of the lack of the genetic testing. Although HCM associated with VSD is rare in adults [1], the present case characterized by the incomplete spontaneous closure of muscular VSD in adults supports for its existence.

With increased availability of cardiac image modalities such as cardiac magnetic resonance imaging (cMRI), detection of left ventricular wall abnormalities, like myocardial clefts, is becoming more common [8–10]. Published data reveal a strong relationship between myocardial clefts and HCM. Indeed, from the morphological point of view, the crypts in the ventricular septal myocardium look similar as the VSDs with incomplete spontaneous closure. The etiology and pathogenesis of these myocardial structural abnormalities remain unclear. Taking our case into consideration, the myocardial crypts (clefts) in the interventricular septum (or part of them) could be the remnants of VSDs during spontaneous closure due to the following reasons. At first, the spontaneous closure of muscular VSD could be due to hypertrophy of the septal myocardium and/or fibrous tissue hyperplasia, etc. [7], which have been confirmed by anatomic evidence from a newborn infant with muscular VSD. In the case, fibrotic tissue replaced the defect, but gross examination revealed a small depression on the interventricular septum’s left side [11]. Secondly, Dasgupta S [12] proposed another possible mechanism for closure of muscular VSD: right ventricular endocardial tissue proliferation and coverage. In the report, a 4 mm midmuscular VSD with a left-to-right shunt in a 2-month-old female infant was observed by echocardiography. Three years later, a repeated echocardiography showed the defect’s right ventricular side was closed, but there was still a 4 mm defect in the left ventricular side. Left-to-right shunt communicating with the right ventricular cavity was not observed. This is very similar to our case. At last, Tikanoja T has reported that the incidence of muscular VSD was higher in children with HCM, but rare in adults based on clinical evidences [5]. However, these clinical observations could not be excluded from the possibility of septal myocardial hypertrophy in adults, apart from the spontaneous closure of most VSDs. With the development of modern diagnostic instruments, the remnants of these VSDs with incomplete spontaneous closure (right ventricular side closure only) shall be more easily detected.

To our knowledge, we are the first to report the HCM case accompanied with incomplete spontaneous closure of muscular VSD and anomalous RCA arising from left sinus of Valsalva, which were revealed through the combination of transthoracic 2D, color Doppler, Contrast-enhanced echocardiography and CTA. Based on our observation, HCM associated anomalies could be easily missed if examined by the single echocardiography. Therefore, the combined use of transthoracic 2D, color Doppler, contrast-enhanced echocardiography, and CTA are necessary to identify the associated congenital abnormalities of HCM.

## Abbreviations

2D: 2-dimensional
CTA: Computed tomography angiography
ECG: Electrocardiogram
HCM: Hypertrophic cardiomyopathy
RCA: Right coronary artery
TTE: Transthoracic echocardiography
VSD: Ventricular septal defect
